# Gastric Cancer Mesenchymal Stem Cells Inhibit NK Cell Function through mTOR Signalling to Promote Tumour Growth

**DOI:** 10.1155/2021/9989790

**Published:** 2021-06-29

**Authors:** Shuwei Guo, Chao Huang, Fengfeng Han, Bin Chen, Ying Ding, Yuanyuan Zhao, Zhihong Chen, Shaodi Wen, Mei Wang, Bo Shen, Wei Zhu

**Affiliations:** ^1^School of Medicine, Jiangsu University, Zhenjiang, Jiangsu, China; ^2^Department of Gastrointestinal Surgery, Affiliated People's Hospital of Jiangsu University, Zhenjiang, Jiangsu, China; ^3^Department of Oncology, Jiangsu Cancer Hospital Affiliated to Nanjing Medical University, Nanjing, Jiangsu, China

## Abstract

The dysfunction of natural killer (NK) cells has been increasingly reported in malignancies, especially in solid tumours. Mesenchymal stem cells (MSCs) exhibit pleiotropic functions that include mediating immune cell exhaustion which is implicated in cancer progression. However, the association of MSCs derived from gastric cancer (gastric cancer mesenchymal stem cells: GCMSCs) with the dysfunction of NK cells remains poorly understood. In this study, we demonstrated that GCMSCs effectively contributed to the exhaustion of NK cells through the release of soluble factors. Furthermore, passivation of the antitumour effect in NK cells was closely associated with their dysfunctional state. The GCMSC-conditioned medium prevented the frequency and effector function of infiltrating NK cells in tumour-bearing mouse models, thus promoting tumour growth. Mechanistically, mammalian target of rapamycin (mTOR) signalling, a critical regulator of cellular metabolism that mediates the function of immune cells, was inhibited in NK cells treated with GCMSCs. However, the checkpoint receptor PD-1 was still present at minimal levels with or without GCMSCs. The study results revealed that GCMSCs contributed to dysfunctional NK cells involved at least partially in the inhibition of mTOR signalling, suggesting potential directions for NK cell-based cancer immunotherapy.

## 1. Introduction

Gastric cancer (GC) is the fifth most commonly diagnosed cancer and the fourth leading cause of cancer death worldwide [1]. Despite major advances in surgical procedures and other therapies, the overall 5-year survival rate for GC remains less than 30% in most countries [2]. The development and progression of GC are influenced by the crosstalk between a tumour and the host immune system [3]. Clinical trials targeting immune checkpoints, such as the PD-1/PD-L1 inhibitory axis, have reported outstanding results for various tumours. However, the objective response rate in GC was only 15% [4]. Therefore, developing an in-depth understanding of the underlying mechanism of immune escape during GC development and progression is essential.

Natural killer (NK) cells are potent cytotoxic innate immune cells with established roles in immune surveillance against tumour cells and viral infection [5]. Many tumour types exhibit a high incidence of major histocompatibility complex (MHC) loss or low neoantigen burden [6], rendering tumour cells refractory to recognition by CD8^+^ T cells. Unlike CD8^+^ T cells, NK cells prefer to mediate non-MHC-restricted killing of tumour cells, which makes them attractive for cancer therapy. Mounting evidence in human cancer studies suggests that NK cell frequency, infiltration, and function improve patient survival [7–9]. One study demonstrated that the percentage of gastric tumour-infiltrating NK cells was negatively correlated with tumour size, invasion, overall survival, lymph node and distant metastasis, and neural invasion status [10]. Another report revealed that the efficacy of PD-1 and PD-L1 blockade relies on the antitumour activity of NK cells [11]. In addition, blockade of the checkpoint receptor TIGIT prevents NK cell exhaustion and elicits potent antitumour immunity [12].

Numerous studies have demonstrated that infiltrating NK cells in malignancies, especially in solid tumours, showed dampened function and changed phenotype [13, 14]. The efficacy of strategies based on NK cells for treating solid tumours remains unsatisfactory [15], indicating the critical restriction of NK cell activity in the immunosuppressive tumour microenvironment (TME). Bian et al. demonstrated that tumour cell metabolically impair T-cell function and tumour immunity by outcompeting T cells for methionine [16]. Tumour and tumour-associated cells produce and secrete various immunosuppressive factors to inhibit NK cell activation, including interleukin (IL)-6, IL-10, transforming growth factor-*β* (TGF-*β*), and prostaglandin E2 (PGE2) [17]. Another study showed that lactate and low pH in the TME reduced the cytotoxic activity of NK cells, leading to decreased tumour killing ability [18]. An increasing number of studies have demonstrated that the TME causes tumour-infiltrating NK cell dysfunction through multiple mechanisms, leading to tumour immune escape.

Mesenchymal stem cells (MSCs) are one of the key cell components of the TME [19]. Our previous studies have demonstrated that MSCs derived from GC tissues (GCMSCs) upregulated PD-L1 expression in GC cells, resulting in tumour escape [20]. In addition, MSCs possess systemic immunoregulatory and immunosuppressive properties and influence both adaptive and innate immune responses directly or through the secretion of soluble molecules [21–24]. We determined that GCMSCs exhibited immunosuppressive potential and increased the proportion of regulatory T cells in peripheral blood mononuclear cells (PBMCs) [25]. Researchers have also paid extensive attention to the effect of MSCs on NK cells. One experiment confirmed that MSCs derived from human lung cancer can cause NK cell dysfunction through PGE2 [26], and bone marrow-derived MSCs enhanced the function of NK cells and increased the release of perforin and granzymes [27]. This result suggested that MSCs from various tissues may exhibit various regulatory functions.

The effect of MSCs from GC on NK cells remains unclear. To address this research gap, we comprehensively and rigorously explored the effects of GCMSCs on NK cell activity. We identified a marked reduction in the effector function molecules and cytotoxicity against tumour cells of NK cells after treatment with GCMSCs. Furthermore, the frequency with which infiltrating NK cells were identified in subcutaneous tumours in mice and their function were both significantly reduced in vivo. Our observations provide insights into the responses of GCMSCs to cancer growth and how they indirectly blunt NK cell activity.

## 2. Materials and Methods

### 2.1. Mice

NOD/ShiLtJGpt-Prkdcem26Cd52Il2rgem26Cd22/Gpt (NCG mice; age: 4–8 weeks) were purchased from Nanjing GemPharmatech. All mice were bred in a special pathogen-free facility for use according to the guidelines for experimental animals at the Jiangsu University. All experiments were approved by the Animal Care and Use Committee of Jiangsu University.

### 2.2. Cell Lines and Cell Culture

The human GC cell lines HGC-27 and MKN-45 were purchased from the Chinese Academy of Sciences Type Culture Collection Committee Cell Bank (Shanghai, China) and were cultured in RPMI-1640 (Biological Industries) with 10% foetal bovine serum (FBS, Biological Industries). NK92 was gifted by BinZhou Medical University in Shandong and cultured in SuperCultureTM L-500 (SL51903, DAKEWE) with 10% FBS and IL-2 (100 IU/ml, PeproTech). All cells were maintained at 37°C in a 5% CO_2_ incubator.

GCMSCs were isolated from GC tissues, which were obtained from GC patients who underwent operations at the Affiliated People's Hospital of Jiangsu University (Zhenjiang, Jiangsu, China). The research was approved by the Ethics Committee of Jiangsu University. GCMSCs were isolated as previously described [28] and were cultured in MEM-ALPHA medium (Biological Industries, Israel) supplemented with 10% FBS.

### 2.3. Multilineage Differentiation Potential of MSCs

Primary GCMSCs were cultured in 12-well plates with MSC medium supplemented with 10% FBS. For induction of osteogenic and adipogenic differentiation, GCMSCs were cultured until 80% confluence was reached, after which they were treated with the MSC Osteo-Staining kit (C37C00150, VivaCell BIOSCIENCES) and MSC Adipo-Staining kit (C37A00150, VivaCell BIOSCIENCES). After 2 weeks of culture, the cells were fixed and stained with oil red O (adipocytes) and Alizarin red S (osteocytes). Calcium deposition and the lipid droplet formation in the cells were determined through microscopy.

### 2.4. Animal Models

NCG mice were intraperitoneally transplanted with 1 × 10^7^ healthy human PBMCs. After 7 days, flow cytometry was performed to determine the proportion of human CD45^+^ cells to lymphocytes in whole blood. Mice with more than 25% of hCD45^+^ cells to lymphocytes in blood were selected. These mice were then injected with 1 × 10^6^ HGC-27 cells subcutaneously, and this was followed by an injection of GCMSC-CM the next day. The GCMSC-CM was dosed to paratumour every other day for 14 days. Tumour growth was monitored and measured using a caliper every other day. Tumour volume was calculated as 0.5 × length × width × width. The mice were euthanised when tumours grew to larger than 1000 mm^3^. After euthanasia, tumours were removed and mechanically dissociated to a size as small as possible in RPMI-1640. A single-cell suspension was isolated by dissociating the tumour tissue in the presence of collagenase IV (C1860, Solarbio) and DNAse I (D8070, Solarbio) for 1 h. The suspension was then strained through a 50 *μ*m filter. The collected cells were used for flow cytometry.

### 2.5. NK Cell Isolation

PBMCs from healthy donors were separated through Ficoll density gradient centrifugation. Enriched NK cells were isolated from the PBMCs using MACS bead (Miltenyi Biotec), and conjugated CD56 antibody labelled mononuclear cells were separated from unlabelled cells through a column in the presence of a magnetic field. Freshly isolated NK cells were immediately used or cultured in L500 medium.

### 2.6. Flow Cytometry and Antibodies

Cells were prepared in a single-cell suspension and washed with phosphate-buffered saline. After incubation with FcR Blocking Reagent (miltenyi, 130-059-901) for 10 min, NK cells were stained with fluorescence antibodies for 30 min at 4°C. The following antibodies were used in this research (all antibodies from BioLegend unless otherwise indicated): FITC-conjugated antibody to human CD3 (300306), CD56-PE (304606), CD107a-APC (328620), PD-1-APC (BD Pharmingen, 558694), Perforin-APC (353311), IFN-*γ*-APC (502512), CFSE, and PI (BD Pharmingen). GCMSCs were assessed for membrane expression of selected markers by using CD19-FITC, CD29-PE, CD34-FITC, CD45-FITC, CD90-PE, and CD105-PE antibodies (all antibodies above from eBioscience). For intracellular cytokine staining, NK cells were stimulated for 5 h with a 2 *μ*l/ml cell stimulation cocktail (plus protein transport inhibitors) (eBioscience) containing phorbol 12-myristate 13-acetate, ionomycin, brefeldin A, and monensin. Then, cells were stained for surface markers, fixed, and permeabilised with eBioscience FoxP3 fixation buffer according to the manufacturer's instructions. The fixed cells were then stained with perforin, CD107a, and IFN-*γ*.

### 2.7. Cytotoxicity Assay

NK cells were collected following treatment with GCMSC-CM. Then, CFSE-labelled target cells (HGC-27, MKN-45) were cocultured with the collected NK cells at different effector to target ratios (1 : 1, 5 : 1, 10 : 1, and 20 : 1) at 37°C in a 5% CO_2_ incubator for 6 h. For spontaneous death control, the CFSE-labelled target cells were cultured alone under the same conditions. Then, PI was added, and lysed cells (CFSE^+^ PI^+^) were identified through flow cytometry.

### 2.8. Proliferation Assay

NK cells were seeded (2 × 10^5^ cells/well) in a 96-well. Following treatment with GCMSC-CM for 48 h, CCK-8 solution (Beyotime Biotechnology) was added to each well. After a 2 h incubation, the absorbance of the experimental and control wells was measured at 450 nm with an automatic microplate reader (BioTek, Winooski, VT, USA).

### 2.9. Apoptosis Assay

The NK92 cells were treated with GCMSC-CM. In this series of experiments, the included controls were cultured using two mixed nutrient solutions (SuperCultureTM L-500 and MEM-ALPHA medium) at a ratio of 1 : 1. Apoptotic rates were evaluated 48 h after the treatment. In accordance with the manufacturer's recommendations (BD Pharmingen), 5 × 10^5^ cells were collected in each tube and 1 ml of annexin V binding buffer was added, followed by thorough mixing. Subsequently, 5 *μ*l of annexin V–FITC was added. After mixing, the tube was incubated in the dark at 4°C for 15 min, after which 10 *μ*l of PI was added. Immediately after, flow cytometry analysis was performed (Beckman Coulter).

### 2.10. Western Blotting

Whole-cell extracts of NK and GCMSC cells were prepared with RIPA buffer containing protease and phosphatase inhibitors (KeyGEN BioTECH). SDS-PAGE gels were used to resolve the cell lysates, and proteins on gel were transferred to PVDF membranes (Millipore, Germany); this was followed by blockade with 5% skimmed milk for 1 h at room temperature. Membranes were incubated with primary antibodies against TGF-*β*, S6/p-S6, GAPDH, and *β*-actin (all antibodies from Abcam) overnight at 4°C. Protein bands were developed through chemiluminescence autoradiography.

### 2.11. Statistical Analysis

Data are presented as the mean ± standard error of the mean. Significance between two groups was determined using the two-tailed paired *t* test in Prism 7 (GraphPad). Other statistics are shown in figure legends. A paired *t* test was used to analyse the differences in NK cell function with and without GCMSC treatment. An unpaired *t* test was used to analyse the difference in infiltration of NK cells in tumours in mice. The Kruskal–Wallis H test was used to analyse differences in tumour growth in vivo. Statistical parameters are presented in the legend of each figure. *P* < 0.05 was considered significant.

## 3. Results

### 3.1. Characterisation of MSCs Isolated from Patients with GC

**Figure 1:**
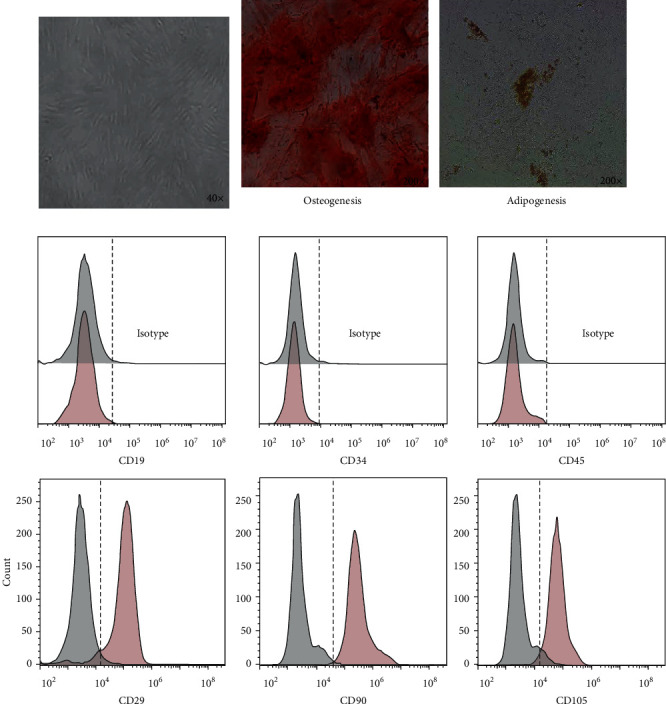
Characterisation of GCMSC. (a) Representative image of GCMSC. (b) Osteogenic differentiation (Alizarin red S, 200x) and adipogenic differentiation (Oil red O, 200x). (c) GCMSC markers (CD19, CD29, CD34, CD45, CD90, and CD105) were analysed through flow cytometry.

The phenotypic and functional features of MSCs isolated from dissociated GC tissues were verified. On day 14, long spindle-shaped fibroblastic cells were observed on MSCs that adhered to the plastic surface (Figure 1(a)). After induction in the conditioned medium, the GCMSCs underwent differentiation to osteocytes and adipocytes in vitro (Figure 1(b)). In addition, antibody staining was performed for flow cytometry. GCMSCs were positive for CD105, CD90, and CD29 but lacked the expression of CD19, CD34, and CD45 (Figure 1(c)).

### 3.2. GCMSCs Effectively Attenuated the Degranulation Ability of NK Cells In Vitro

**Figure 2:**
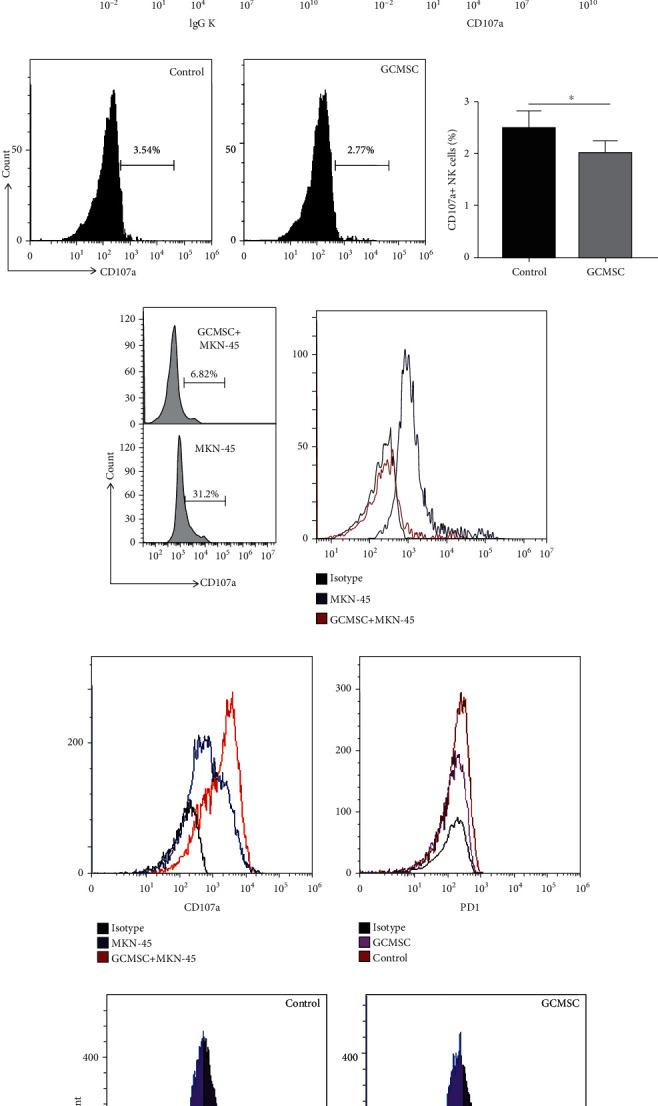
GCMSCs directly attenuated the degranulation ability of NK cells. (a) FCM gating strategy for enriched NK cells (CD56^+^) from the peripheral blood of healthy donors. (b) Representative histograms of CD107a in freshly isolated NK cells. (c) NK cells cocultured with or without GCMSCs at a 1 : 1 ratio for 48 h were analysed through flow cytometry for CD107a expression. (d) Quantification of CD107a expression in NK cells shown in (c). Data are presented as mean ± SEM. Paired *t* test, ^∗^*P* < 0.05. (e) Analysis of CD107a+ NK cells. Production of CD107a in response to GCMSCs followed by stimulation with MKN-45 for 5 h was measured. (f) Enriched NK cells cocultured with MKN-45 cells with or without GCMSC for 7 days. Representative histograms of CD107a and PD-1 are shown. (g) NK92 cells cocultured with GCMSCs for 48 h. Representative histograms of CD107a are shown. (h) Quantification of CD107a expression. Data are presented as mean ± SEM. Paired *t* test, ^∗∗^*P* < 0.01. (i) NK92 cells cocultured with HGC-27 and/or GCMSCs. Representative histograms of CD107a in NK92 cells are shown.

Given the immunosuppressive potential of MSCs in the TME, we explored the relationship between GCMSCs and NK cells. Enriched NK cells (CD56^+^ cells) from the peripheral blood of healthy donors were cocultured with GCMSCs for 48 h, after which degranulation ability (CD107a expression) was assessed through flow cytometry. The results revealed that NK cells from different donors displayed similar but minimal levels of CD107a expression, and the levels were more reduced in the presence of GCMSCs than in the control (Figures 2(a)–2(d)), which suggests that GCMSCs weakened the degranulation function of NK cells. We then found that GCMSCs more strongly suppressed degranulation following activation by MKN-45 (Figure 2(e)). Another analysis revealed that GCMSCs inhibited degranulation for a long coculture time but could not induce higher PD-1 expression levels in NK cells (Figure 2(f)). A similar finding for suppression by GCMSC was also confirmed in NK92 cells (Figures 2(g)–2(h)). To further confirm this phenomenon, NK92 cells were cocultured with GCMSCs or HGC-27 separately or in combination. GCMSCs effectively reversed the activation of NK92 by GC cells (Figure 2(i)). These results revealed that treatment with GCMSCs attenuated the degranulation ability of NK cells.

### 3.3. GCMSCs Significantly Induced an Exhaustion State in NK Cells in an Indirect Manner

**Figure 3:**
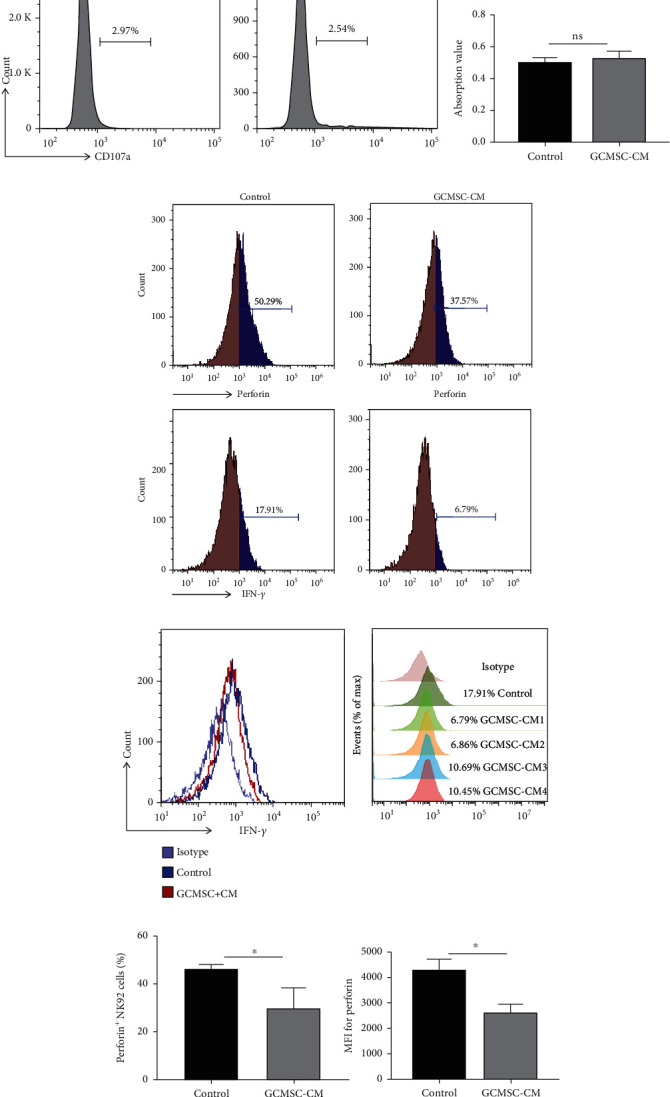
GCMSC-CM effectively induced an exhaustion state in NK cells. (a) NK92 cells treated with GCMSC-CM for 48 h were analysed through flow cytometry for CD107a. (b) Quantification of the percentage of positive cells shown in (a). (c) Analysis of CD107a expression in NK cells isolated from healthy donors cultured with or without GCMSC-CM through flow cytometry. (d) Quantification of the effect of GCMSC-CM on NK cell proliferation by CCK8. (e, f) Representative FCM plot of the indicated molecules in NK92 cells treated with GCMSC-CM. Results are representative of three independent experiments. (g) Quantification of the percentage and mean fluorescence intensity (MFI) of the indicated molecules shown in (e, f). (b, d, and g) Data are shown as mean ± SEM. A paired *t* test was used for comparisons. ^∗^*P* < 0.05, ^∗∗^*P* < 0.01.

NK cells were treated with supernatant derived from GCMSCs (GCMSC-CM). The degranulation ability (CD107a expression) of NK92 cells was significantly reduced compared with control groups (Figures 3(a)–3(b)), whereas NK cells from healthy donors exhibited slightly lower expression (Figure 3(c)). Given the homology and safety of animal experiments, we focused on the influence of GCMSCs on NK cells through the secretion of soluble factor. We determined that GCMSC-CM could not effectively promote or inhibit the proliferation potential of NK92 cells (Figure 3(d)). In addition, NK92 cells treated with GCMSC-CM were stained with annexin V and PI for apoptosis assay through flow cytometry. The results revealed that GCMSC-CM could not significantly induce apoptosis compared with the control (Figure S1A–1B). Next, other markers associated with degranulation and cytokine production of NK cells were characterised. Consistent with previous results, the expression of perforin and IFN-*γ* was inhibited after treatment with GCMSC-CM, suggesting impaired degranulation and reduced cytokine production in NK cells (Figures 3(e)–3(g)). These results indicated that GCMSCs indirectly induced a dysfunctional state in NK cells without affecting their proliferation potential.

**Figure 4:**
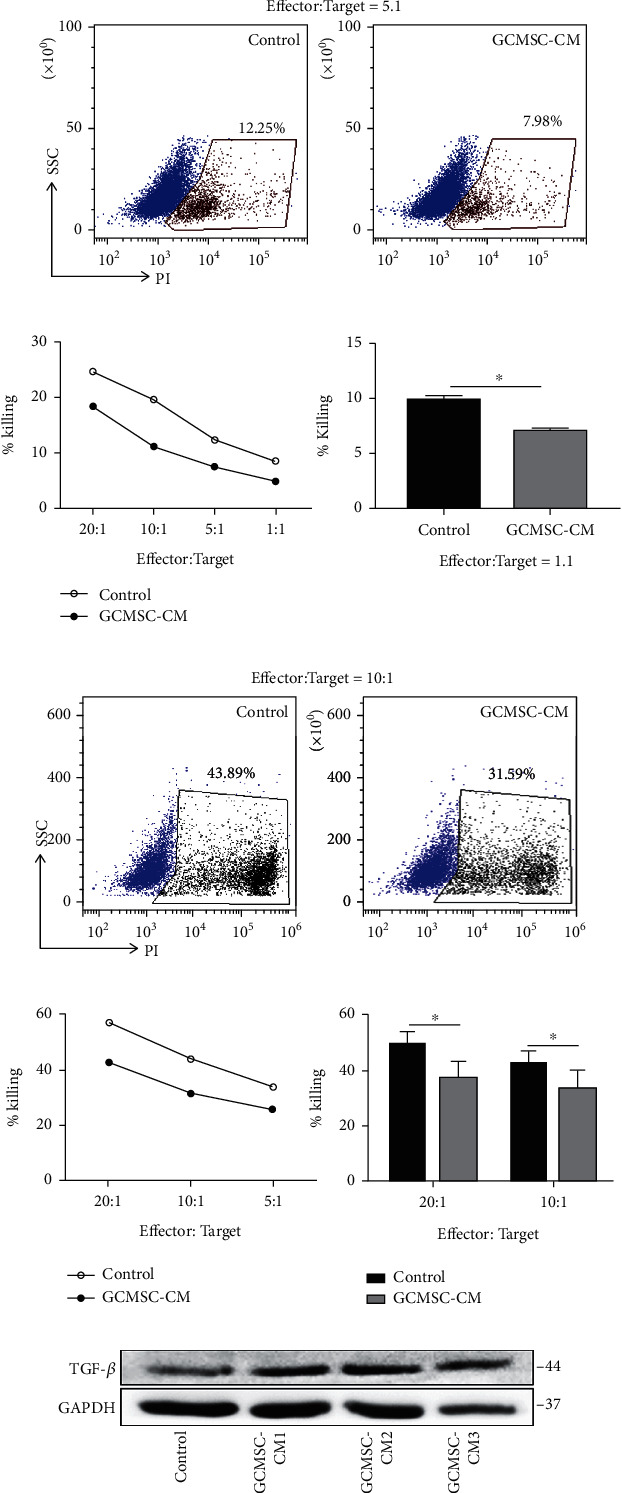
Attenuated cytotoxicity of NK cells after treatment with by GCMSC-CM. (a) Cytotoxicity against MKN-45 targets by NK92 cells previously treated with or without GCMSC-CM. Representative FCM plot of PI expression in target cells. (b) Six-hour cytotoxicity assay of NK92 cells against MKN-45 cells. Cytotoxicity of NK92 against MKN-45 cells at the indicated ratios (NK cells/target cells) (left). Quantification of the cytotoxicity assay at a ratio of 1 : 1 (right). (c) Cytotoxicity against HGC-27 target cells by NK cells derived from healthy donors. Representative FCM plot of PI expression in target cells. (d) Six-hour cytotoxicity against HGC-27 cells of NK cells pretreated with GCMSC-CM. Cytotoxicity of NK cells against HGC-27 cells at the different ratios (left). Quantification of the cytotoxicity assay at the indicated ratios (right). (e) NK 92 cells were treated with GCMSC-CM for 48 h, and TGF-*β* expression was evaluated through immunoblotting.

To further assess the effector functions of NK cells, their cytotoxicity was examined. When cocultured with MKN-45 cells, NK92 cells treated with GCMSC-CM exhibited significantly attenuated cytotoxicity compared with untreated cells (Figures 4(a)–4(b)). Similar results were noted for NK cells in the PBMCs of healthy donors (Figures 4(c)–4(d)), confirming the attenuated cytotoxicity of NK cells. In addition, we found that the expression of the known immunosuppressive cytokine TGF-*β* increased in NK cells following treatment with GCMSC-CM (Figure 4(e)), which may have affected the antitumour potential of other immune cells and further weakened antitumour immunity in the TME. These results comprehensively indicated that NK cells treated with GCMSC-CM also exhibited a diminished cytotoxicity ability against tumour.

### 3.4. GCMSC-Treated NK Cells Exhibited Attenuated Antitumour Activity In Vivo

**Figure 5:**
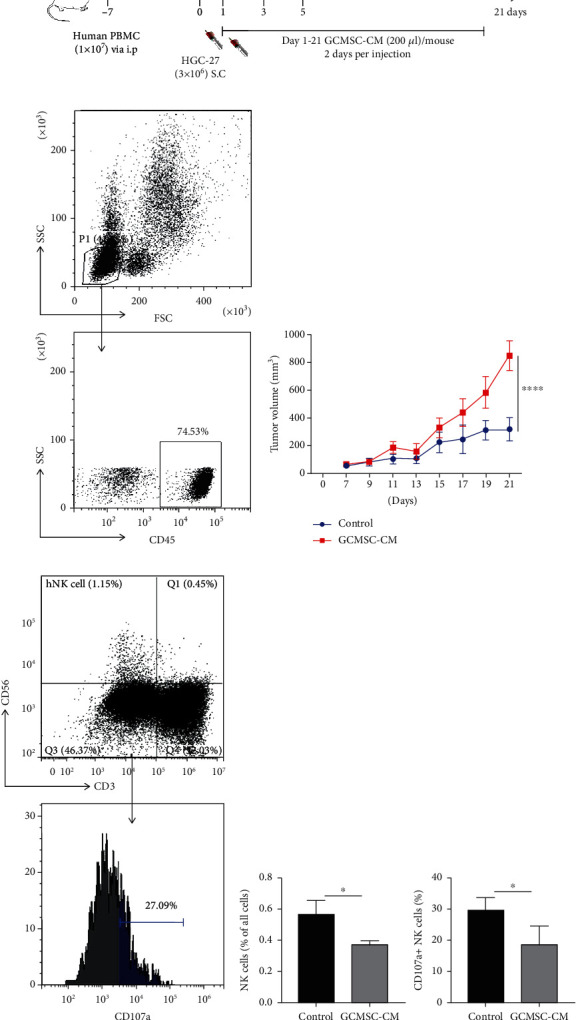
GCMSC-treated NK cells exhibit attenuated antitumour activity in vivo. (a) In vivo treatment scheme. NCG mice were injected with 1 × 10^7^ PBMCs from healthy donors. The successfully constructed model mice was selected for the following experiment 7 days later. (b) Representative flow cytometry plot of human CD45^+^ cells in mouse populations from peripheral blood 7 days after PBMC treatment. (c) Tumour volumes (mean ± SEM) in mice at various time points after the challenge are shown. Kruskal–Wallis *H* test, ^∗∗∗∗^*P* < 0.0001. (d) Representative flow cytometry plot of infiltrating NK cells (top) in tumour and histogram of CD107a (bottom) expression in infiltrating NK cells. (e) Quantification of the frequency of infiltrating NK cells and the expression of CD107a in intratumoural NK cells. Data are presented as mean ± SEM. Unpaired two-tailed *t* test, ^∗^*P* < 0.05.

To evaluate the antitumour potential of NK cells treated with GCMSC-CM in vivo, we assessed their ability to kill GC cells in a mouse xenograft tumour model. Mice (NCG) were administered with human PBMC cells to mimic the human immune system. The mice with more than 25% of human CD45^+^ cells to lymphocytes in whole blood were selected and were injected with HGC-27 cells subcutaneously; this was followed by a subcutaneous injection of GCMSC-CM the next day. A dose of GCMSC-CM was administered every other day for 14 days to influence the NK cells (Figures 5(a) and 5(b)). Tumour growth was monitored every other day. PBMC exhibited significantly higher antitumour activity compared with the GCMSC-CM group (Figure 5(c)), which suggests that the antitumour function of immune cells in PBMC was suppressed. We also investigated the frequency and number of infiltrating human NK cells (hCD3^−^CD56^+^cells). The frequency and degranulation ability (CD107a) of the infiltrating NK cells were significantly attenuated as evidenced by flow cytometry (Figures 5(d) and 5(e)), which indicates that the suppression of the antitumour function of immune cells in PBMCs by the supernatant likely depends on NK cells. Together, these results confirm that GCMSC-CM can dampen the antitumour potential of NK cells in an indirect manner in vivo.

### 3.5. GCMSCs May Contribute to the Exhaustion of Infiltrating NK Cells in Human GC

To investigate the function and frequency of NK cells in human GC, mRNA expression was analysed using the ONCOMINE database (http://www.oncomine.org). The mRNA expression of CD56 was significantly higher in normal gastric tissues than in GC tissues (Figure S2A) [29], which was consistent with our results obtained from limited clinical samples (Figure S2B). Based on the TNM stage classification of GC, the data from the database revealed that the proportion of NK cells decreases gradually along with tumour progression (Figure S2C) [30]. We collected 244 human GC samples from The Cancer Genome Atlas (TCGA) database and analysed the overall survival of CD56 in GC patients; the results revealed that higher CD56 expression did not mean longer or better overall survival (Figure S2D). These results suggested an exhaustion and decreased frequency of NK cells in human GC. To explore the effect of GCMSCs on NK cells in human GC, we performed regression analysis between GCMSC markers and CD56. The analysis of data from TCGA database showed that GCMSC markers have a direct linear correlation with CD56 (Figure S2E), which further verified the potential interaction between NK cells and GCMSCs in humans. These results indicated that GCMSCs likely contribute to the dysfunction and decreased frequency of infiltrating NK cells even in human GC with tumour progression.

### 3.6. Attenuated Effector Function in NK Cells Is Partly Mediated by mTOR Signalling

**Figure 6:**
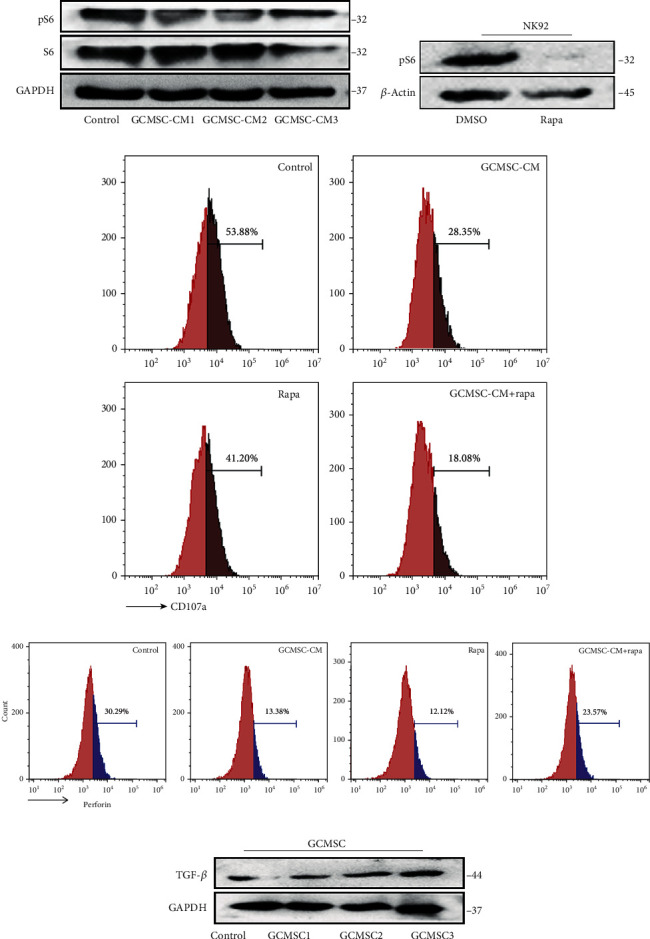
Attenuated effector function in NK cells is partly mediated by mTOR signalling. (a) Western blot analysis of mTOR downstream activation (pS6) in NK92 cells cultured in GCMSC-CM for 48 h. (b) pS6 expression in NK92 cells was detected after treatment with or without rapamycin (100 ng/ml) for 48 h by using a Western blot. (c, d) NK92 cells were incubated with rapamycin and GCMSC-CM. Representative histograms of indicated molecules of CD107a and perforin are shown. (e) Indirect Transwell cocultures between GCMSCs and NK92 and TGF-*β* expression in GCMSC were evaluated using Western blot. GCMSCs derived from three different patients.

Cellular metabolism is critical to immune cell function. We investigated whether the reduction in NK cell function by GCMSCs depended on mTOR signalling, a key regulator of cellular metabolism. We determined that mTOR signalling activity was downregulated in GCMSC-CM-treated NK92 cells, as indicated by a decreased level of phosphorylated S6 (p-S6; Figure 6(a)). In addition, treatment with rapamycin (rapa) alone, an inhibitor of mTOR1, significantly diminished the effector function of NK92 cells, as indicated by reduced levels of CD107a and perforin (Figures 6(b)–6(d)). To further verify whether mTOR signalling plays a critical role in the exhaustion of NK cells treated with GCMSCs, we evaluated the effector function after treatment with GCMSC-CM and/or rapa. We observed that either GCMSC-CM or rapa effectively downregulated the effector molecules, and the degranulation ability was further decreased under treatment with a combination of GCMSC-CM and rapa (Figure 6(c)). This result indicated that GCMSCs attenuate NK cell function at least partially through mTOR signalling.

TGF-*β*, a known immunosuppressive factor of NK cell function, is produced by GCMSCs. Moreover, when cocultured with NK92 cells, GCMSCs expressed high levels of TGF-*β* (Figure 6(e)). These results suggest that GCMSCs may influence NK cell function in more than one way.

## 4. Discussion

In humans, infiltrating NK cells in tumours influence disease progression and patient survival, and a higher frequency of NK cells often indicates better overall survival [31]. In solid tumours, however, tumour-infiltrating NK cells frequently exhibit a dysfunctional state [12, 32, 33]. One study demonstrated that the percentages and numbers of tumour-infiltrating NK cells were in a functional impairment state and were decreased in human GC [10]. We obtained the same results in a single cell suspension of human GC tissue through flow analysis (Figure S2). Tumour cells evade NK cell-mediated surveillance by developing an immunosuppressive microenvironment in which NK cell dysfunction is caused by the crosstalk between NK cells and tumour, stromal, and immune cells.

As a critical component in the TME, MSCs can effectively promote the initiation and promotion of many types of tumours [34]. MSCs participate in the immune modulation of the TME and promote tumour immune escape [19]. They have also been reported to participate in immune regulation and to exert immunosuppressive effects on T cells. We questioned whether GCMSCs were also involved in the functional regulation of NK cells. Galland et al. demonstrated that lung tumour-derived MSCs effectively reduce NK cell function and modulate NK phenotype through soluble factors in vitro [26]. However, considering the heterogeneity of TME among various types of tumours, MSCs may have different effects on NK cells. To our knowledge, this is the first investigation of the effect of GC-derived MSCs on NK cells.

In the present study, GCMSCs significantly attenuated the degranulation ability (CD107a) of NK cells isolated from the peripheral blood of healthy donors. Moreover, NK92 cells treated with GCMSCs showed a similarly impaired state. These results suggest that GCMSCs are likely to be a key factor influencing NK cell dysfunction in the TME. To further confirm this phenomenon, we cocultured tumour cells with GCMSCs and NK cells to simulate the TME, and the results revealed that tumour cells can further activate NK cells, but this activation can be reversed by GCMSCs. These results indicate that GCMSCs effectively limit the antitumour immunity of NK cells in vitro. When treated with GCMSC-CM, both degranulation and cytokine production (CD107a, perforin, and IFN-*γ*) were significantly attenuated compared with the untreated group either in peripheral blood NK cells or NK92 cells. Furthermore, NK cells pretreated with GCMSC-CM exhibited significantly lower cytotoxicity against tumour cells, indicating that GCMSCs can inhibit NK cell function by secreting soluble factors. Studies have reported the elevated expression of TGF-*β*, indoleamine-2, 3-dioxygenase, and PGE2, which suppress immune cell function, in the TME [35, 36]. Whether the immunosuppressive effect of GCMSCs contributed to these factors remains to be confirmed.

We found that GCMSCs cocultured with NK cells expressed higher levels of TGF-*β* compared with GCMSCs alone, suggesting that TGF-*β* may be a critical factor inhibiting NK cell function. Unexpectedly, we found that GCMSCs can also upregulate the level of TGF-*β* in NK cells, which contributed to the formation of a systemic immunosuppressive microenvironment. Moreover, cellular metabolism plays a critical role in regulating the function of immune cells [37, 38], but data on the ability of MSCs to dampen NK cell activity via impaired metabolism are scarce. Also unexpectedly, the mTOR signalling of NK cells after GCMSC-CM treatment was significantly inhibited, and simultaneously, the effector function of NK cells after rapa treatment was significantly weakened, which indicates that dampened NK cell function is at least partially due to the inhibition of mTOR. However, our results indicated that after mTOR is inhibited by rapa, the degranulation ability (CD107a) of NK cells was further impaired by GCMSC-CM, suggesting that mTOR is not the only pathway through which GCMSCs affect NK cells. Considering the strong immunomodulatory properties of MSCs through ligand-receptor binding in a cell contact-dependent manner or through the secretion of various soluble factors [39], GCMSCs attenuate NK cell function through various mechanisms. Immunotherapy targeting the PD-1/PD-L1 inhibitory axis elicited long-term remission in a broad spectrum of cancers [11]. Moreover, PD-1 expression on human NK cells has recently been reported in several cancer indications, including GC [40–42]. However, our results revealed that PD-1 levels in freshly isolated peripheral blood NK cells are minimal, indicating that the NK cells recruited into the tumour are educated by the TME. GCMSCs could not upregulate the PD-1 level in NK cells in vitro, which was consistent with the finding of minimal PD-1 expression in mouse and human NK cells under multiple conditions [43]. A more thorough understanding of the expression of inhibitory markers is critical to advance the clinical application of NK immunotherapy.

Finally, regarding subcutaneous tumours in mice, we selected the immune system of mice reconstituted with healthy human PBMC cells. Our results indicated that GCMSC-CM drastically reduced the frequency of infiltrating NK cells as well as attenuated their degranulation ability, which might also contribute to tumour growth. Moreover, the lower frequency tumour infiltrating NK cells may be a consequence of either a decreased capacity of NK cells to migrate from the peripheral blood or enhanced apoptosis of NK cells that infiltrate tumours. NK cells have been reported to be sensitive to H_2_O_2_-induced apoptosis [44]. The results of the present study indicate that GCMSC-CM cannot induce the proliferation or apoptosis of NK cells in vitro. However, the GCMSC-CM treatment group exhibited a decreased number of infiltrating NK cells, which means that GCMSCs effectively influenced the infiltration of NK cells. Moreover, studies have reported that MSCs affect the function of effector T cells. Our previous research found that GCMSCs could upregulate PD-L1 expression in tumor cells, which resulted in the resistance of tumor cells to CD8^+^ T cells cytotoxicity [20]. Therefore, whether GCMSCs also affect T cells and lead to tumour immune escape remains unclear, which is the limitation of our research. This study focused more on the critical role of dampened NK cells involved in immune escape.

## 5. Conclusions

In summary, our findings provide a new framework for understanding the relationship between GCMSCs and NK cells in GC. In the present study, GCMSCs reduced the frequency and function of GC-infiltrating NK cells, resulting in the immune escape of GC cells. A more in-depth investigation of the target of GCMSCs affecting NK cells is urgently needed. Overall, restoring the function of NK cells by blocking the crosstalk between NK cells and GCMSCs as well as through checkpoint blockade therapy could be a useful strategy for preventing GC immune escape.

## Data Availability

The data used to support the findings of this study are included within the article.

## Supplementary Materials

Supplementary Materials Supplementary Figure 1: GCMSC-CM could not effectively induce NK92 cell apoptosis. (a) Effects of GCMSC-CM on NK92 apoptosis. Representative FCM plot of indicated molecules in NK92 cells. (b) Quantification of the percentage of apoptotic cells shown in (a). Supplementary Figure 2: GCMSCs were involved in the exhaustion of infiltrating NK cells in human gastric cancer. (a) CD56 expression (NK cells) of GC tissues and adjacent normal gastric tissues (ONCOMINE database). Paired two-tailed *t* test, ^∗∗∗∗^*P* < 0.0001. (b) Representative FCM plot of NK cells (CD3^−^CD56^+^) in gastric cancer tissues (intratumoral (IT) regions) and corresponding adjacent normal tissues (peritumoral (PT) regions) from three different patients. (c) Correlation between CD56 expression and gastric cancer stage (TNM); T: tumor; N: node (regional lymph node); M: metastasis. Patients who were in more advanced stages tended to express lower CD56 expression (ONCOMINE database). (d) Survival of low and high expression of CD56 in gastric cancer patients. Mantel-Cox test. (e) Correlation analysis between GCMSC markers (CD90 and CD105) and CD56 expression in GC tissues (Supplementary Materials).
