# Supplementary material for: Gastric Cancer Mesenchymal Stem Cells Inhibit NK Cell Function through mTOR Signalling to Promote Tumour Growth
Source: Stem Cells Int. 2021 Jun 29;2021:9989790. doi: 10.1155/2021/9989790 (PMC8263240; doi:10.1155/2021/9989790)
Supplement: Supplementary Materials — Supplementary Figure 1: GCMSC-CM could not effectively induce NK92 cell apoptosis. (a) Effects of GCMSC-CM on NK92 apoptosis. Representative FCM plot of indicated molecules in NK92 cells. (b) Quantification of the percentage of apoptotic cells shown in (a). Supplementary Figure 2: GCMSCs were involved in the exhaustion of infiltrating NK cells in human gastric cancer. (a) CD56 expression (NK cells) of GC tissues and adjacent normal gastric tissues (ONCOMINE database). Paired two-tailed t test, ∗∗∗∗P < 0.0001. (b) Representative FCM plot of NK cells (CD3−CD56+) in gastric cancer tissues (intratumoral (IT) regions) and corresponding adjacent normal tissues (peritumoral (PT) regions) from three different patients. (c) Correlation between CD56 expression and gastric cancer stage (TNM); T: tumor; N: node (regional lymph node); M: metastasis. Patients who were in more advanced stages tended to express lower CD56 expression (ONCOMINE database). (d) Survival of low and high expression of CD56 in gastric cancer patients. Mantel-Cox test. (e) Correlation analysis between GCMSC markers (CD90 and CD105) and CD56 expression in GC tissues (Supplementary Materials). [file 9989790.f1.pdf]

Gastric cancer mesenchymal stem cells inhibit NK cell function through mTOR signaling to promote tumor growth

Shuwei Guo[1], Chao Huang[1], Fengfeng Han[1]， Bin Chen[1], Ying Ding[1], Yuanyuan Zhao[1], Zhihong Chen[3], Shaodi Wen[2], Mei Wang[1], Bo Shen[2], Wei Zhu[1*]

1. School of Medicine, Jiangsu University, Zhenjiang, Jiangsu, China
2. Department of Oncology, Jiangsu Cancer Hospital Affiliated to Nanjing Medical University, Nanjing, Jiangsu, China
3. Department of Gastrointestinal Surgery, Affiliated People's Hospital of Jiangsu University, Zhenjiang, Jiangsu, China

Corresponding author: Wei Zhu, PhD. School of Medicine, Jiangsu University, 301 Xuefu Road, Zhenjiang, Jiangsu, 212013, China. E-mail: zhuwei@ujs.edu.cn.

Supplementary Fig. S1

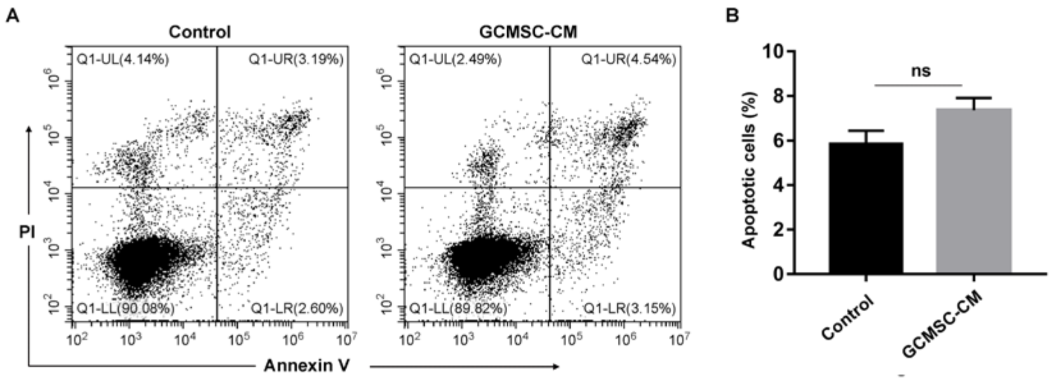

Supplementary Fig. S1 GCMSC-CM could not effectively induce NK92 cell apoptosis. (A) Effects of GCMSC-CM on NK92 apoptosis. Representative FCM plot of indicated molecules in NK92 cells. (B) Quantification of the percentage of apoptotic cells shown in A.

Supplementary Fig. S2

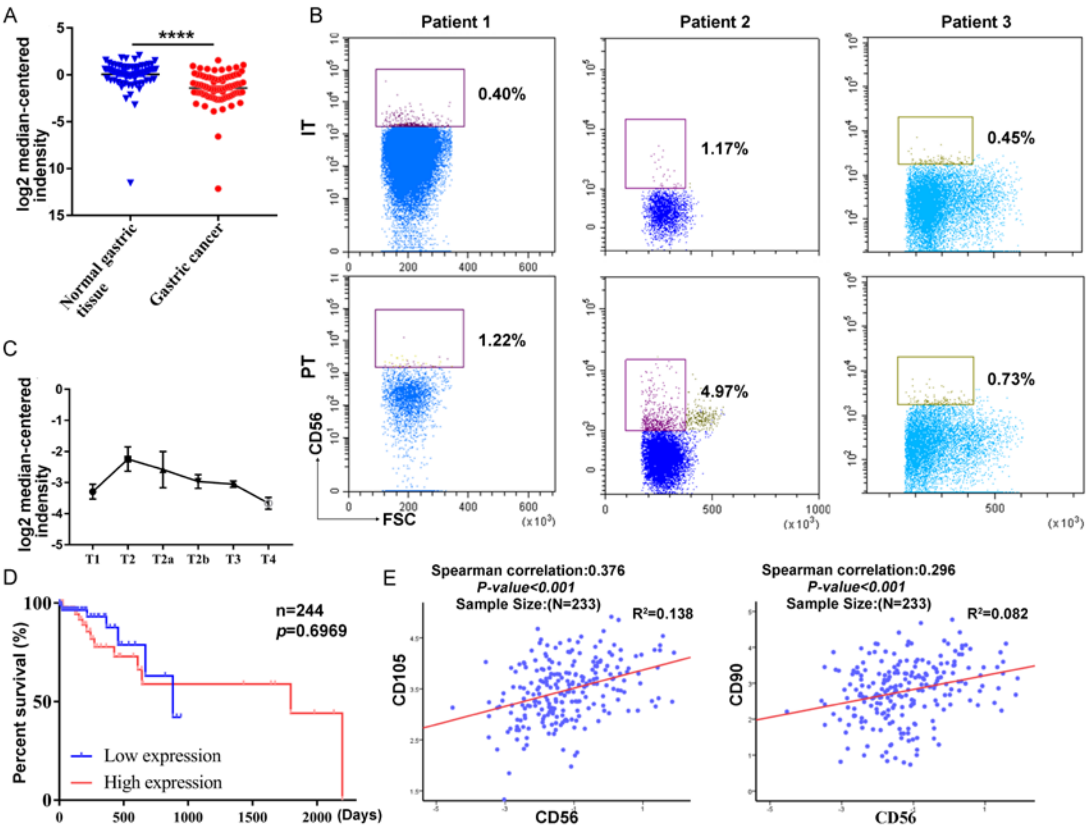

Supplementary Fig. S2 GCMSCs were involved in the exhaustion of infiltrating NK cells in human gastric cancer

(A) CD56 expression (NK cells) of GC tissues and adjacent normal gastric tissues (ONCOMINE database). Paired two-tailed *t* test, *****P*<0.0001. (B) Representative FCM plot of NK cells ($CD3^-CD56^+$) in gastric cancer tissues (Intratumoral (IT) regions) and corresponding adjacent normal tissues (Peritumoral (PT) regions) from three different patients. (C) Correlation between CD56 expression and gastric cancer stage (TNM), T—Tumor， N—Node (regional lymph node), M—metastasis. Patients who were in more advanced stages tended to express lower CD56 expression (ONCOMINE database). (D) Survival of low and high expression of CD56 in gastric cancer patients. Mantel-Cox test. (E) Correlation analysis between GCMSC markers (CD90 and CD105) and CD56 expression in GC tissues.
